# MicroRNAs and Recent Insights into Pediatric Ovarian Cancers

**DOI:** 10.3389/fonc.2013.00095

**Published:** 2013-04-30

**Authors:** Jessica C. Francis, Nonna Kolomeyevskaya, Claire M. Mach, Jennifer E. Dietrich, Matthew L. Anderson

**Affiliations:** ^1^Department of Obstetrics and Gynecology, Baylor College of MedicineHouston, TX, USA; ^2^Department of Gynecologic Oncology, Roswell Park Cancer InstituteBuffalo, NY, USA; ^3^College of Pharmacy, University of HoustonHouston, TX, USA; ^4^Department of Pathology and Immunology, Baylor College of MedicineHouston, TX, USA; ^5^Dan L. Duncan Cancer Center, Baylor College of MedicineHouston, TX, USA

**Keywords:** microRNA, DICER1, FOXL2, sex cord-stromal tumors, granulosa cell tumors, ovarian cancer, germ cell tumors

## Abstract

Ovarian cancer is the most common pediatric gynecologic malignancy. When diagnosed in children, ovarian cancers present unique challenges that differ dramatically from those faced by adults. Here, we review the spectrum of ovarian cancers found in young women and girls and discuss the biology of these diseases. A number of advances have recently shed significant new understanding on the potential causes of ovarian cancer in this unique population. Particular emphasis is placed on understanding how altered expression of non-coding RNA transcripts known as microRNAs play a key role in the etiology of ovarian germ cell and sex cord-stromal tumors. Emerging transgenic models for these diseases are also reviewed. Lastly, future challenges and opportunities for understanding pediatric ovarian cancers, delineating clinically useful biomarkers, and developing targeted therapies are discussed.

## Introduction

Adnexal masses are a common cause for the medical evaluation of young women and girls. Approximately 5–10% of all women in the United States undergo surgery for an adnexal mass at some point in their lives (Barakat et al., 2009). In most cases, adnexal masses in young women are benign cystadenomas that arise either from the ovary or developmental remnants of paratubal tissue. However, 2–8% pediatric adnexal masses are ultimately diagnosed as cancer (Barakat et al., 2009).

## Clinical Spectrum

In contrast to adult ovarian cancers, pediatric ovarian cancers typically originate in germ cells or stroma rather than epithelia lining the ovarian surface, distal fallopian tube, or peritoneal implants of endometriosis. Approximately 40% of ovarian tumors are germ cell tumors whereas 25% are sex cord-stromal tumors (Barakat et al., 2009). Germ cell ovarian cancers are a diverse category of tumors that include both benign and malignant disease. Ovarian teratomas (“dermoids”) are the most common and perhaps best-known example of a benign ovarian germ cell tumor. While their true incidence in the general population is unknown, dermoids account for approximately 65% of adnexal masses in pediatric patients presenting for treatment (Ehren et al., 1984). Malignant ovarian germ cell tumors are much less common than their benign counterparts. Histologically, malignant germ cell tumors recapitulate rudimentary tissues observed during normal human development (Table 1). The most common malignant ovarian germ cell tumor is dysgerminoma (Chieffi et al., 2012). Other germ cell tumors observed in the ovary include immature teratomas and endodermal sinus tumors. Combinations of different histologic elements are frequently observed in the same ovarian tumor. These “mixed” tumors frequently contain elements of dysgerminoma (Chieffi et al., 2012).

**Table 1:** **Classification of pediatric ovarian tumors**.

**GERM CELL TUMORS**
Teratoma
Dysgerminoma
Yolk-sac tumor
Embryonal carcinoma
Polyembryoma
Choriocarcinoma
Mixed germ cell tumor
**SEX CORD-STROMAL TUMORS**
Granulosa-stromal cell tumors
Adult type
Juvenile type
Thecoma-fibroma tumors
Thecoma
Fibroma-fibrosarcoma
Sclerosing stromal tumor
Sertoli-stromal cell tumors
Sertoli cell tumor
Leydig cell tumor
Retiform
Mixed
Sex cord tumor with annular tubules
Unclassified
Gynandroblastoma

Ovarian sex cord-stromal tumors are also relatively common in the pediatric population (Table 1) (Barakat et al., 2009). Granulosa cell tumors (GCTs) are the most common sex-cord-stromal tumor. GCTs account for 2–5% of these malignancies regardless of age at diagnosis. Ovarian GCTS cancers are categorized as either juvenile or adult type. Juvenile granulosa ovarian cancers (JGCT) occur mainly in premenarchal girls and comprise roughly 5% of GCTs (Gell et al., 1998). Adult type granulosa cell cancers (AGCT) tend to occur in older women but can also occur in girls and young women. The JGCT and AGCT subtypes are histologically distinguished by the fact that cells in AGCT exhibit “coffee bean” nuclei and are often organized into fluid filled structures known as Call-Exner bodies. Similar features are seen only rarely in JGCT (Schneider et al., 2005).

Pediatric ovarian cancers frequently present with symptoms, such as premature menarche, that can be attributed to their ability to secrete estrogen and other biologically active substances. This feature reflects the origin of GCTs and sex cord-stromal tumors in cells involved in the synthesis and secretion of steroid hormones. However, benign ovarian cysts can also be associated with specific estrogen-induced findings, including the premature development of breast tissue, onset of menstruation, and clitoromegaly. In one study of 34 patients with precocious puberty, ovarian cysts were responsible for pubertal physical findings in 7% of young girls (Mitrovic et al., 2006). Depending on the hormonal activity of a granulosa cell or Sertoli–Leydig tumor, patients may present with contrasexual rather than isosexual precocious puberty. Isosexual precocious puberty in young women is accompanied by elevated levels of serum estrogen as well as a failure to observe a gonadotropin response following GnRH stimulation. Contrasexual precocious puberty is associated with androgen secretion, results in virilization and can usually be attributed to Sertoli–Leydig tumors.

## Inherited Syndromes

The causes of ovarian cancer in both the adult and pediatric populations are not yet well understood. However, significant advances have been recently made in delineating molecular events that underlie inherited syndromes of ovarian cancer in both the adult and pediatric populations. In adults, epithelial ovarian cancers have been associated with dominant negative mutations in gene products (BRCA1/2) that play a critical role in DNA damage repair (Pruthi et al., 2010). Recent advances in understanding the biology of BRCA1/2 have led to the discovery that many high grade serous ovarian cancers likely originate either in the distal fallopian tube or at an epithelial transition between the fallopian tube and ovary. (Auersperg et al., 2008; Crum et al., 2012).

Currently, there is no evidence to indicate that mutations associated with epithelial ovarian cancers in adults are causally linked to pediatric ovarian cancers. Instead, pediatric ovarian cancers are associated with a number of hereditary and non-hereditary syndromes distinct from the syndromes of breast/ovarian cancer linked to BRCA1/2. The first syndrome shown to be associated with pediatric ovarian cancer is Ollier disease. Ollier disease is a non-hereditary disorder of the skeleton that is characterized by cartilaginous implants (enchondromas) in the metaphyseal portion of long bones (Gell et al., 1998). The estimated prevalence of Ollier disease in the U.S. population is roughly 1 per 100,000. Nine cases of juvenile granulosa cell tumor (JGCT) of the ovary have been reported in individuals with Ollier disease (Rietveld et al., 2009), a number that is significantly greater than expected by chance. Very little is currently known about the mechanisms linking Ollier disease and ovarian cancer. However, it has been noted that the ovarian tumors that develop in patients with Ollier disease are usually unilateral and are frequently noted on the same side of the body as the patient’s most recently resected endochondroma (Gell et al., 1998).

Pediatric ovarian cancers have also been reported as part of the disease spectrum for inherited syndromes of pleuropulmonary blastoma (PPB), multinodular goiter (MNG), and blepharophimosis-ptosis-epicanthus inversus syndrome (Crisponi et al., 2001; Doros et al., 2012). Both PPB and MNG have been previously linked to the same region on human chromosome 14q, suggesting a shared genetic etiology (Bignell et al., 1997). PPB is a rare pediatric mesenchymal thoracic tumor that results in lesions histologically characterized by immature epithelium and mesenchymal cells resembling fetal lung. While the exact incidence of ovarian tumors in patients with familial PPB is unknown, ovarian sex-cord-stromal tumors appear to be a relatively infrequent manifestation of this syndrome. One recent study has reported that 9/325 individuals with this syndrome had been diagnosed with an ovarian sex cord-stromal tumor (Schultz et al., 2011).

**Figure 1:**
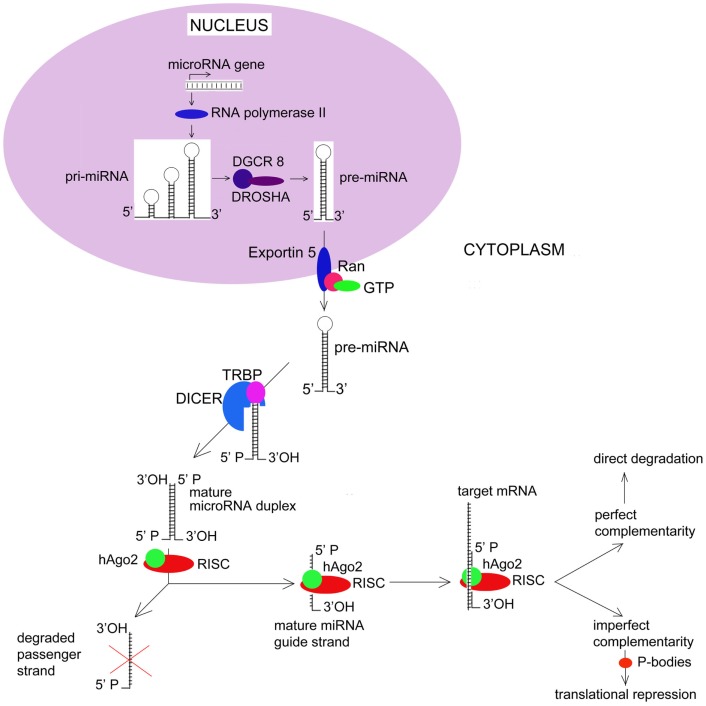
**MicroRNA Biogenesis and Function**. Most human miRNAs are initially transcribed as long precursors known as pri-miRNAs. Pri-miRNAs undergo a series of processing events that ultimately result in the cytoplasmic release of a mature double-stranded miRNA. Key events in miRNA biogenesis include active export of pre-miRNAs from the nucleus via a nuclear membrane complex that includes RANGAP1 and RANBP1. Once within the cytoplasm, pre-miRNAs undergo endonucleolytic cleavage by DICER1. Recent evidence now suggests that passenger miRNA strands may actively target patterns of gene expression rather than undergoing degradation. hAGO2, human Argonaut; RISC, RNA-induced Silencing Complex.

Germline mutations in *DICER1*, a key endonuclease involved in microRNA (miRNA) processing (see Figure 1), have been identified in a nearly one-half of children diagnosed with PPB, including PPB patients previously diagnosed with an ovarian sex-cord-stromal tumors (Hill et al., 2009). In most cases, the reported *DICER1* mutations are predicted to generate a truncated protein with impaired function. Similar germline mutations in *DICER1* have also been identified in familial syndromes of MNG (Rio Frio et al., 2011). Like PBB, a subset of familial MNG patients develops ovarian Sertoli–Leydig cell tumors, a type of ovarian sex cord-stromal tumor (see Table 1). However, in contrast to the situation with PPB, the penetrance of Sertoli–Leydig cell tumors in MNG patients with *DICER1* mutations appears to be much higher. This higher penetrance likely reflects the distinct spectrum of mutations discovered in the MNG population. MNG-associated mutations include both missense mutations and in-frame deletions. In particular, the spectrum of DICER1 mutations in families with MNG have been predicted to selectively disrupt the PAZ domain of this gene product, impacting the RNase domains of DICER1 responsible for trimming pre-miRNAs to their mature length (Lau et al., 2009; Rio Frio et al., 2011). Existing evidence suggests that these mutations are also associated with an earlier age at onset for Sertoli–Leydig cell tumors.

## Molecular Biology

A wide range of other strategies in addition to traditional pedigree studies have been used to advance our understanding of pediatric ovarian cancers (Table 2). Similar to the work discussed above, this work points to a key role for non-coding RNA transcripts known as microRNAs (miRNAs) in these diseases. Other studies have implicated a transcription factor known as FOXL2. Many aspects of the mechanisms by which disruptions in the miRNA expression or FOXL2 function lead to ovarian cancer remain poorly understood.

**Table 2:** **Recently identified genes of interest in pediatric ovarian cancers**.

Germ cell tumors	Sex cord-stromal tumors	Borderline tumors
TSPY (human)	DICER1 (human)	KRAS (human)
c-kit (human)	Gct1 (mouse)	BRAF (human)
Inhibin-alpha (mouse)	Wnt (mouse/human)	PIK3CA (human)
SMAD1/5 (mouse)	FOXL2 (human)	EGFR (human)
	PI-3 kinase (mouse/human)	PDGFRA (human)
	mTORC1 (mouse/human)	CTNNB1 (human)

### DICER1 and Sertoli–Leydig cell tumors

MicroRNAs are small, highly conserved non-coding RNA transcripts that play a critical role in silencing patterns of gene expression (Kasinski and Slack, 2010). As genetic elements, miRNAs have been previously implicated in the regulation of cellular differentiation, proliferation, and apoptosis and have been shown to function as both tumor suppressors and oncogenes (Zhang et al., 2007). Aberrant miRNA expression has been demonstrated in various cancers of hematologic, gastrointestinal, genitourinary, pulmonary, endocrine, neurologic, and dermatologic origin. (Zhang et al., 2006; Creighton et al., 2010; Medina et al., 2010; Trang et al., 2010; Iorio and Croce, 2012).

The human genome contains approximately 1000 miRNAs. Of interest, the genomic loci for more than 50% of miRNAs are found within the cancer associated genomic regions (CAGR), fragile sites, regions of loss of heterozygosity, amplification, and viral integration sites (Zhang et al., 2006). Generally, overexpression of miRNAs is the result of gene amplification, deregulation of a transcription factor, or demethylation of CpG islands in the promoter region. In contrast, miRNA down-regulation is guided by gene deletions, epigenetic silencing, and loss of transcription factor expression (Corney et al., 2007; Zhang et al., 2007; Mirnezami et al., 2009; Ruan et al., 2009). One of the proposed mechanisms of cancer metastasis is promoter CpG island hypermethylation that blocks the expression of tumor suppressor miRNAs (Lujambio et al., 2008). In turn, decreased miRNA expression impacts genes responsible for angiogenesis, invasion, and cell adherence. These include cadherins, tissue inhibitors of metalloproteinases, and thrombospondins (Wiemer, 2007).

MicroRNA genes are typically located in the introns of protein-coding genes, where they are transcribed by RNA polymerase II either independently or as part of a polycistronic cluster (Bartel, 2004). Initial transcription of most miRNAs results in the creation of a long primary transcript, known as the pri-microRNA (pri-miRNA). While still within the nucleus, pri-miRNAs are 5′-capped and 3′-polyadenylated, after which, they are processed by a 650-kDa microprocessor complex that includes the ribonuclease III (RNase III) Drosha and the dsRNA-binding protein DGCR8. Pri-miRNA processing by Drosha results in a 70-nucleotide hairpin structures known as pre-microRNAs that are actively transported to the cytoplasm. Cytoplasmic export is an energy-dependent process that relies on a nuclear membrane complex and involves multiple gene products including Exportin 5 (XPO5) and multiple guanine nucleotide exchange factors such as RANBP1 and RANGAP1. Once within the cytoplasm, pre-miRNAs are cleaved by the RNase III endonuclease DICER1. This final processing step involves the transactivating response RNA binding protein (TRBP), and results in the release of a mature, double-stranded miRNA molecule.

Functionally, miRNAs can regulate gene expression by a number of different mechanisms. In most cases, these involve direct binding between nucleotides located within the miRNA “seed” and complementary sequences in the 3′-untranslated region (3′-UTR) of its target mRNA. In the case of perfect complementary, endonucleolytic cleavage of the mRNA occurs. This is thought to be the primary mechanism by which miRNAs result in translational inhibition. The ability of miRNAs to induce mRNA degradation has been shown to depend on their ability to incorporate mRNAs into the RNA-induced silencing complex (RISC). The RISC is a multiprotein complex that also contains human argonaute family of proteins – hAgo2 – that have endonuclease activity. These proteins have RNA-binding domains – PAZ and PIWI that resembles ribonuclease H; they bind to single-stranded 3′ end of the miRNA and 5′ end of the miRNA guide strand. After the passenger strand of the miRNA is degraded, the remaining guide strand of the mature miRNA directs the RISC complex to the 3′-UTR of target mRNA.

Alternatively, imperfect complementary between a miRNA and its target mRNA results in translation repression (Saraiya et al., 2013). Processing bodies (P-bodies) are recently discovered structures that store target mRNAs away from ribosomes. There, target mRNAs undergo enzymatic degradation by decapping enzymes Dcp1/Dcp2, 5′–3′ exonuclease Xrn1, and de-adenylating enzymes. Thus miRNAs down-regulate expression of target genes at the level of translation. Current understanding of key events in miRNA biogenesis and biology are summarized in Figure 1.

As discussed above, mutations in *DICER1* have been implicated in inherited syndromes of pediatric ovarian cancer. In mammalian cells, DICER1 plays a key role in final cytoplasmic processing of pre-miRNAs leading to the release of the mature miRNA. Recently, investigators have reported that somatic mutations in *DICER1* are frequently observed in ovarian sex cord-stromal tumors (Heravi-Moussavi et al., 2012). These mutations were found in as many as 60% of Sertoli–Leydig cell tumors, most frequently impact nucleotides encoding Asp1709 or Glu1813. These investigators found similar mutations in *DICER1* only rarely in other solid human tumors, such as endometrial and ovarian cancers (Heravi-Moussavi et al., 2012). In contrast to the germline mutations in *DICER1* reported in PPB and MNG, the somatic *DICER1* mutational “hot-spots” identified in Sertoli–Leydig cell tumors are located at metal-binding sites critical for miRNA interaction and cleavage within the RNase IIIb domain of this gene product. They are predicted to have little or no impact on DICER1’s RNase IIIa activity, meaning that DICER1 function in these patients is disrupted rather than lost.

How *DICER1* mutations impact the miRNA repertoire of the cells and tissues in which they are expressed in an important question that has yet to be resolved. Profiling of tumors and lymphoblastoid cell lines derived from patients with MNG have found evidence for decreased expression of miR-345, let-7a, miR-99b, miR-133, and miR-194 in common between MNG and normal-appearing thyroid tissue of patients carrying one of these mutations. Of these, only miR-345 is highly expressed in normal thyroid; let-7a has not been previously implicated in thyroid disease (Rio Frio et al., 2011). Mutations in *DICER1* altered the RNase IIIb activity of *DICER1* have also been hypothesized to generate a bias toward the processing or selection of transcripts that primarily depend on RNase IIIa activity, such as 3′ mature miRNAs. Until recently, these transcripts, previously known as miR* strands, were thought to largely lack biologic function (Aktas et al., 2013). In general, miRNA transcripts located on the 5′ arm of miRNA precursors (5′ mature miRNAs) were expressed at much greater abundance in many cells and tissues. A number of other observations have also suggested that 5′ mature miRNAs are preferentially loaded by *DICER1* into the RISC and that this capacity does not depend on the integrity of its RNase IIIa domain (Risch et al., 1996; Noland et al., 2011). However, it is now clear that 3′ mature miRNAs (miR* strands) are also able to target patterns of gene expression in seed-dependent fashion similar to miRNA guide strands (Yang et al., 2011). Recent *in vitro* evidence generated with genetically modified clones of DICER1 has confirmed that mutations impacting the RNase IIIb domain of *DICER1* result in a near complete loss of 5′ mature miRNAs with little impact on the expression of 3′ mature miRNAs. Remarkably, mutations in the RNase IIIa domain of DICER1 had the opposite effect, enriching the populations of miRNA found in cells for 3′ mature transcripts. Thus, altering the miRNA landscape by selective mutations in a specific domain of *DICER1* could easily lead to an oncogenic environment with distinct tissue specificities.

### FOXL2 and granulosa cell tumors

Significant progress has also been made in understanding the molecular basis of human ovarian GCTs using high throughput sequencing. These studies indicate that juvenile and adult granulosa cell tumors may be molecularly distinct, despite the fact that they can share clinical and histologic features. Using whole-transcriptome paired-end RNA sequencing, Shah et al. (2009) recently identified a somatic missense mutation (C to G) at position 412 of *FOXL2*, a member of the forkhead-winged-helix family of transcription factors, in four human ovarian GCTs. *FOXL2* is a transcription factor that plays an important role in female reproductive tract development and function in both mouse and humans (Cocquet et al., 2002). In humans, highest levels of FOXL2 expression are found in ovarian granulosa cells with lower levels of expression also observed in ovarian stroma. Robust FOXL2 expression is also observed in the human eyelid.

In granulosa cells, FOXL2 functions as a transcriptional repressor, regulating the expression of key gene products important for granulosa cell function in adult ovary, including steroidogenic acute regulatory protein (STAR), P450 aromatase and cyclin D2 (Kuo et al., 2012). In mice, *Foxl2* represses the expression of FSH-dependent genes in granulosa cells (Garcia-Ortiz et al., 2009). Its disruption results in premature ovarian failure (Uda et al., 2004). In humans, FOXL2 has been shown to have a role in regulating ovarian transcription of gonadotropin-releasing hormone receptor as part of an AP1-SMAD3-SMAD4 complex (Ellsworth et al., 2003). This latter observation is noteworthy in that transforming growth factor β (TGF-β) regulates granulosa cell proliferation in large part by activating SMAD2 and SMAD3 via its cell surface receptor. Defects in TGF-β signaling regulated by crosstalk between ovarian germ cells and stroma are also commonly observed in ovarian germ cell tumors, which will be discussed below.

As mentioned above, germline mutations in *FOXL2* have been described in families with blepharophimosis-ptosis-epicanthus inversus syndrome (Crisponi et al., 2001). This observation is significant, because ovarian GCTs occur at an increased rate in a subset of families with this syndrome. Subsequent characterization of additional specimens identified the same mutation in 86/89 adult granulosa cell tumors (AGCTs), but only 1/10 JGCTs and 3/14 benign ovarian thecomas. Notably, no evidence for this mutation was found in 49 specimens representative of other types of ovarian sex-cord-stromal tumors or 329 unrelated ovarian or breast tumors of different histologies.

The mechanisms by which position 412 (C to G) mutations disrupt FOXL2 function and leads to an ovarian sex cord-stromal tumor are not yet clear. The amino acid residue altered by this mutation is located on the surface of the forkhead DNA-binding domain characteristic of this transcription factor, where it appears to interfere with normal transcriptional activity (Shah et al., 2009). At least one recent study has found that the patterns of gene expression in human GCTs are highly enriched for FOXL2 transcriptional targets (Benayoun et al., 2012). However, the mechanisms by which clinically relevant mutations in *FOXL2* impact transcription in granulosa or other cells have yet to be precisely demonstrated. Molecular modeling suggests that this missense mutation fails to disrupt folding of DNA-binding domain or its ability to interact with DNA (Shah et al., 2009). Furthermore, no differences were observed in the intracellular localization of mutant FOXL2. Thus, the mutation associated with GCTs would appear to promote tumorigenesis by other mechanisms, possibly by altering FOXL2’s capacity to interact with one or more of its multiple binding partners. It is also possible that *FOXL2* mutations cooperative with other genetic events even though the protein products generated by these genes do not directly interact with one another. For example, monosomy 22, gains of chromosomes 12, 14, and loss of X have all been reported as recurrent abnormalities in both adult and JGCTs in humans (Fletcher et al., 1991). The mechanisms by which these latter genomic imbalances promote the initiation or progression of GCTs are not known. Although they do not appear to contribute to a loss of FOXL2 expression in *FOXL2* wild-type AGCT, it is possible that the altered patterns of gene expression they induce may have a permissive effect for tumorigenesis in the background of FOXL2 mutations or by altering activity in pathways normally regulated by this transcription factor (Geiersbach et al., 2011).

### Mouse models of granulosa cell tumors

Due to the relative rarity of pediatric ovarian cancer, obtaining adequate tissue samples for research purposes can be difficult. Over the course of the past 20 years, a number of mouse models for ovarian GCTs have been developed that are potentially useful for understanding these cancers. Granulosa cells are the most proliferative cells found in the adult mammalian ovary. Perhaps reflecting this proliferative capacity, ovarian GCTs are relatively common in many mammalian species. GCTs of the ovary occur spontaneously in a number of specific inbred strains of mice. At least one of the loci (*Gct1*) responsible for these tumors has been mapped to a 1.31 × 10^6^ base pair interval on mouse chromosome 4 (Smith et al., 2012). This region is orthologous to human chromosome 1p36.22 and encodes four known protein-coding gene products. All four of these gene products appear to be expressed in normal mouse and human ovary. One (Tnfrsf8) is a member of the TNF-receptor superfamily known to bind TNF-alpha and regulates both proliferation and apoptosis. Of note, the genomic region at 1p36.22 in humans has been previously implicated in diffuse large B-cell lymphomas (Kreisel et al., 2011) and is frequently lost in both hepatocellular carcinomas (Zhang et al., 2010) and infiltrating ductal carcinomas of the breast (Hawthorn et al., 2010). It is not currently known whether the 1p3.22 locus or any of the genetic elements it encodes contribute to the initiation or metastasis of GCTs. In part, answers to this question have been difficult to obtain, because the relative rarity of human ovarian GCTs has precluded adequately powered genome-wide association studies.

Transgenic mice have also emerged as a potential tool for examining the molecular mechanisms involved in ovarian GCTs. In general, the transgenic events leading to GCTs in mice disrupt key pathways regulating granulosa cell proliferation. The first mouse model for ovarian GCTs was a transgenic knockout of inhibin-alpha (Matzuk et al., 1992). Inhibin-alpha is a key paracrine factor synthesized in granulosa cells that is involved in the normal proliferation of germ cells and somatic cells as well as the assembly of ovarian follicles in both humans and mice. Ovaries from inhibin-alpha knockout mice exhibit early follicular recruitment and advanced follicular growth. These phenotypes are due to a defect in the normal communication between germ cell and somatic cells. In turn, these defects eventually lead to large ovarian tumors (Myers et al., 2009). Conditional deletion of Smad1 and Smad5 in the somatic cells of the ovary of mice has been shown to also result in metastatic tumors (Pangas et al., 2008). Tumor development in these models is suppressed by two functionally distinct receptors for bone morphogenetic protein (BMP) found in murine granulosa cells (Edson et al., 2010). Other genetic lesions that lead to ovarian tumors in mice include the expression of a stable, active mutant from of CTNNB1 that activates wnt/β-catenin signaling and produces dysplastic ovarian lesions that eventually develop into GCTs (Boerboom et al., 2005). Work with these animals has shown that both k-ras activation and PTEN loss enhance CTNNB1-induced ovarian tumorigenesis (Richards et al., 2012). However, mutations that are seemingly unrelated to inhibin signaling pathways also result in GCTs in mice. For example, ovarian tumors have been found in the ovaries of transgenic mice deficient for *Fancf*, a DNA damage repair gene (Bakker et al., 2012).

Importantly, mouse models for GCTs at least partially phenocopy human disease. Hyperactivation of PI-3 kinase signaling and mammalian target of rapamycin complex 1 (mTORC1) are common features of both human and mouse ovarian GCTs. In both healthy women and mice, FSH acts via its receptor on the surface of ovarian granulosa cells to stimulate phosphatidyl inositol 3-kinase (PI3K)-AKT signaling pathways. In mice, an activating mutation (H1047R) in PI3KCA coupled with PTEN loss has been recently shown to be sufficient to induce both ovarian GCTs and serous ovarian adenocarcinomas in mice (Kinross et al., 2012). Both the H1047R mutation and PTEN inactivation were required for early, robust activation of Akt and the development of tumors in these animals. Pharmacologic mTOR inhibition has been shown to delay tumor growth and prolong survival in this model. The investigators responsible for this work went on to examine a series of human ovarian granulosa cell cancers, finding PIK3CA mutations co-existent with both KRAS and PTEN mutations. Other analyses of human GCTs have found increased levels of mTOR protein as well as enhanced expression of the downstream mTOR effectors RPS6KB1, RPS6, eIF4B, and PPARG relative to normal granulosa cells (Kinross et al., 2012). These observations suggest that activity in mTOR pathways are also increased in human GCTs. PI-3 kinase signaling has been recently explored as a potential therapeutic target for human GCTs (Ongeri et al., 2005). However, preclinical data would suggest that mTOR may not be adequate as a therapeutic target in this scenario. When everolimus (an orally administered inhibitor of mTOR) was used to treat a mouse model [Pten (tm1Hwu/tmiHwu); Ctnnb1 (tm1Mmt/+); Amhr2 (tm3(cre)Bhr/+] in which mTOR, RPS6KB1, eIF4B, and PPARG are upregulated in tumor cells similar to human GCTs, investigators were unable to fully reverse the molecular consequences of mTOR activation despite the fact that the use of everolimus slowed tumor growth (Rico et al., 2012). At least one group has questioned the relevance of these findings. Bittinger et al. (2009) recently examined a series of human ovarian GCTs as well as two cell lines (COV434 and KGN), using direct sequencing to screen for mutations. Expression of PIK3CA, PIK3R1, and PTEN was observed in all specimens and neither deletions nor mutations of the exons known to be “hot spots” for these genes could be identified in GCTs.

### Ovarian germ cell tumors

Germ cell tumors account for 15% of malignances diagnosed in both boys and girls during childhood and adolescence. Dysgerminomas of the ovary are analogous to testicular seminomas. These tumors originate from the primordial germ cells (PGC) arrested in their development. Their pluripotency directs further malignant transformation which mimics embryogenesis: embryonal carcinoma (EC), teratoma, yolk-sac tumor (YST), and choriocarcinoma (CH).

The incidence of ovarian dysgerminomas is increasing for reasons that remain unclear (Frazier and Amatruda, 2009). The cell of origin for all germ cell tumors is currently believed to be an early germline progenitor known as a PGC. Pediatric germ cell tumors are widely believed to develop due to dysregulated activity in the embryonic signaling pathways that regulate PGC differentiation and maturation. Key pathways important in this process include both Notch- and Hedgehog-regulated signaling. Multiple components of these pathways have been shown to be aberrantly activated in subsets of pediatric germ cell tumors (Morichika et al., 2010). Perhaps the best studied pathway potentially involved in the pathogenesis of pediatric germ cell tumors is regulated by TGF-β. Multiple components of the TGF-β and BMP families are robustly expressed in developing germ cells and somatic cells in both the male and female gonad (Itman et al., 2006). Significant evidence has implicated aberrant activation of TGF-β signaling in testicular germ cell tumors (Dias et al., 2009) Activation of BMP pathways appears to be most robust in pediatric YSTs (Fustino et al., 2011).

Due to their shared origin in a PGC, the events leading to ovarian and testicular germ cell tumors are widely assumed to be similar. However, it is less clear how widely these observations made in testicular germ cell tumors, such as those described above, actually apply to germ cell tumors occurring in females. An accurate assessment of ovarian germ cell tumors is hindered by the fact that ovarian germ cell tumors occur much less frequently than their testicular counterparts. Furthermore, there appears to be significant inter-tumoral heterogeneity in the activation of these pathways, even within tumors with similar histology (Fustino et al., 2011).

While infrequent, we do know that malignant transformation of germ cells often depends on the presence of genetic material from a Y chromosome (Kota et al., 2012). The most likely candidate gene for dysgerminomas found in the critical region of the Y chromosome responsible for gonadoblastoma is TSPY1 (Chandley and Cooke, 1994). It has long been known that dysgerminomas are frequently are observed in dysgenetic gonads due to the presence of all or a portion of the human Y chromosome. As many as 35 copies of the TSPY1 gene are found on the human Y chromosome (Manz et al., 1993). The gene product generated by *TSPY1* is typically expressed only in testis and appears to be involved in spermatogenesis. High levels of its expression have been observed in gonadoblastomas recovered from dysgenetic patients (Hersmus et al., 2012). However, the mechanisms by which TSPY1 expression and/or the presence of Y chromosomal material potentially leads to ovarian germ cell tumors are unclear. Activating mutations and amplification of the proto-oncogene c-kit, a growth factor receptor, have also been reported to occur in as many as one-third of ovarian germ cell tumors (Cheng et al., 2011). These mutations have been associated with advanced stage disease and may contribute to the progression of this disease. In addition, c-kit has been shown to play an important role in regulating the differentiation of PGCs. Two recent genome-wide association studies have implicated KITLG, the kit ligand, DMRT1, and SPRY3 in familial testicular cancer (Kanetsky et al., 2009; Turnbull et al., 2010). It is possible that similar mechanisms are at work in ovarian germ cell tumors. Lastly, recent work points to a role for dysregulated miRNA expression as a potential cause of ovarian germ cell tumors. Currently the majority of data on miRNA expression in these tumors comes from testicular germ cell tumors. At least one group has reported that the overexpression of miR-371 and miR-302 miRNA families is consistently observed in ovarian germ cell tumors (Palmer et al., 2010). Both miR-371 and miR-302 potentially target a wide variety of gene products relevant to cancer growth and metastasis. This observation is intriguing as a recent genetic screen has identified miRNAs from the miR-371 cluster as putative oncogenes in human germ cell tumors (Voorhoeve et al., 2006). The investigators initially reporting these observations have followed up on this initial report, confirming that the miR-371-373 cluster is highly expressed in seminomas, EC, and YST of both ovarian and testicular origin (Gillis et al., 2007). The biologic role of this cluster appears to be related to wild-type TP53 gene activity in germ cell tumor cells, suggesting that the capacity of this locus to regulate TP53 is a crucial player in the pathogenesis of these tumors. Other potential roles include the capacity of both miR-373 and miR-520c to inhibit CD44, potentially promoting metastatic spread of disease spread. Other work has recently shown that miRNA identified by these studies can also be detected in human serum, opening the door for their potential use as diagnostic and prognostic biomarkers (Murray et al., 2011). This creates new opportunities for evaluating therapeutic responses or monitoring patients for recurrent disease.

## Future Directions

Despite the excitement generated by the recent discovery of *FOXL2* and *DICER1* as key tumor suppressors in pediatric ovarian cancers, there is still much to be understood regarding the mechanisms by which these events lead to cancer. Answers will ultimately prove important not only for understanding ovarian cancer but also many other solid human tumors. For example, a number of recent reports have found that *DICER1* expression is lost in subsets of epithelial ovarian and uterine cancers and that reduced levels of DICER1 play a key role in determining clinical outcomes (Merritt et al., 2008; Zighelboim et al., 2011). Germline *DICER1* mutations have also been described in a number of other rare cancers that occur in children, such cervical embryonal rhabdomyosarcoma and pituitary blastoma (Choong, 2012).

Determining how altered *DICER1* expression and or *DICER1* mutations impact miRNA expression and function as tumor suppressors will be important for assessing how best this insight can be used to develop therapeutic targets and biomarkers. Answers to these questions may come from transgenic mice. Mice with conditional *Dicer1* knockout support the relevance of both *Dicer1* and miRNAs in normal development in diverse tissues, including the female reproductive tract (Nagaraja et al., 2008). Loss of a single *Dicer1* allele does not appear sufficient to induce cancer, despite its ability to reduce time to tumor onset and overall survival murine models of cancer (Kumar et al., 2009). These latter observations seem to indicate that, in mice, *Dicer1* is a haplotype insufficient tumor suppressor. In contrast, the phenotypes associated with somatic *DICER1* mutations in humans suggest these mutations function as a dominant phenotype. However, it will be critical to more precisely examine these questions in greater depth. The initial reports examining *DICER1* studied normal tissues from only a small number of the patients (Kumar et al., 2009). Germline mutations in *DICER1* were observed in four of these specimens, suggesting that *DICER1* mutations in humans conform to the traditional two-hit model of carcinogenesis. Ultimately, resolution of this question will require examining germline DNA in addition to tumor DNA across a much larger panel of patients.

Effective utilization of recent discoveries may also require that we broaden the scope of our search to better understand how altered DICER1 expression and function may play a role in carcinogenesis. A recent study using *Amhr2-Cre/Dicer* *^fl/fl^* mice has shown that complete *Dicer1* loss, when combined with PTEN knockout in Müllerian-derived cells, leads poorly differentiated tumors that in the oviduct (Kim et al., 2012a,b). However, tumors that develop in this model appear to originate in stromal cells of the tube, rather than tubal epithelia, ovarian stroma, granulosa cells, or germ cells. This observation challenges the view that complete loss of *Dicer1* is lethal to tumor cells. It also underscores the need to learn how dysregulated activity in other pathways involved in miRNA biogenesis may be potentially involved in pediatric and other cancers (Yang and Lai, 2011). As discussed above, several of the *DICER1* mutations observed in pediatric ovarian cancers have been shown to disrupt rather than suppress normal patterns of miRNA expression. Understanding how the miRNA landscape is shifted from a tumor suppressive to oncogenic milieu will ultimately be important for determining how the insights discussed above can be best applied clinically.

It will also likely be important to recognize the impact of *DICER1* mutations in pediatric ovarian cancers could be much more complex than a simple gain or loss of function. Recent evidence indicates that disease states can lead to non-templated addition of nucleotides to mature human miRNAs that impact function (Guo et al., 2011). However, when combined with insight into the genetic context in which these alterations lead to cancer, such knowledge would open the door to miRNA-based therapies that could be used in a highly specific and rationale manner to either reverse or prevent these diseases. Similarly, many questions remain unsettled regarding the role of FOXL2 in pediatric ovarian cancers. Perhaps foremost of these is how the mutations identified impact FOXL2 function. Given the central role in forkhead domain transcription factors in regulating diverse functions within normal ovary as well as the proliferation, metabolism, and apoptosis, efforts to dissect the cell-specific role of this transcription factor will likely shed significant insight into normal ovarian development, as well as the mechanisms by which the dysregulated activity of these cells leads to a cancer.

Efforts to explore other mechanisms potentially contributing to the pathogenesis of both ovarian germ cell and sex-cord-stromal tumors should not be neglected. For example, multiple mechanisms could conceivably lead to altered *DICER1* expression or function in pediatric ovarian cancers in addition to recently identified mutations. For example, work examining transcriptional profiles generated for epithelial ovarian cancers by the Cancer Genome Atlas consortium (TCGA) has been unable to confirm that levels of *DICER1* transcript are decreased in epithelial ovarian cancers (Creighton et al., 2012). This observation is curious, as reduced expression of DICER1 protein has notably reported to determine ovarian cancer outcomes (Merritt et al., 2008). This discrepancy could be explained if *DICER1* expression is largely regulated by epigenetic events that suppress translation of *DICER1* mRNA in a subset of cancers, leaving transcript levels unchanged. Similar mechanisms could contribute to the pathogenesis of Sertoli–Leydig cell tumors or other categories of pediatric sex-cord-stromal tumors where germline or somatic *DICER1* mutations have not been observed.

Ultimately, the greatest challenge for utilizing recent insights into the biology of pediatric ovarian cancers will be to create rationale for effective treatments. Attempts to replace the activity of dysfunctional tumor suppressors, such as TP53, have been largely ineffective. For this reason, replacement or targeting of key miRNAs or downstream transcriptional targets whose expression is altered by *DICER1* and *FOXL2* mutations may be the most efficient route to clinical success (Bader et al., 2011). However, even in the case of TP53, a number of recent reports have identified novel approaches to overcome the adverse impact of a dominant dysfunctional gene product (Pruschy et al., 2001). Thus, it may be possible to adopt similar strategies for overcoming cancer-associated mutations in *DICER1* or *FOXL2*.

Even under circumstances where it may not be possible to effectively manipulate DICER1 for adequate therapeutic advantage, it may be possible to target specific miRNAs. One of the important aspects of recent work examining the role of *DICER1* mutations in carcinogenesis has been the discovery that a reduction in DICER1 expression does not uniformly impact the expression of all miRNAs. Thus, it may be possible to identify one or more individual miRNAs whose expression can be specifically targeted for therapeutic purposes in tumors with DICER1 mutations. Although early results have been encouraging, the concept of targeting miRNAs will likely prove to be a significant challenge as well. A number of recent papers have demonstrated that anti-sense miRNA oligonucleotides (AMO) can be used to down-regulate oncogenic miRNAs and silence them (Weiler et al., 2006). Another promising therapeutic approach would be to directly deliver synthetic mimics for key tumor suppressor miRNAs to tumor tissue where the expression of these miRNAs has been lost. Lastly, miRNAs differentially expressed in pediatric ovarian cancers RNA expression profiles can also be potentially diagnostic markers. MiRNA microarray analysis is a valuable technique that highlights aberrant miRNA expression even in poorly differentiated cancer cells. Similarly knowing the expression profile can determine response to therapy, predict patient survival, and distinguish more aggressive or resistant cancer subtypes. Fortunately, the recent insights discussed here have placed us well along the path toward improving outcomes for children diagnosed with one of these cancers.

## Conflict of Interest Statement

The authors declare that the research was conducted in the absence of any commercial or financial relationships that could be construed as a potential conflict of interest.
